# Modified percutaneous iliosacral screw and anterior internal fixator technique for treating unstable pelvic fractures: a retrospective study

**DOI:** 10.1186/s12891-022-06036-8

**Published:** 2022-12-06

**Authors:** Xu-Song Li, Li-Ben Huang, Yu Kong, Meng-Qiang Fan, Yang Zheng, Jie-Feng Huang

**Affiliations:** 1 Department of Orthopaedics & Traumatology, Zhongshan Hospital of Traditional Chinese Medicine, Guangdong Zhongshan, 528401 China; 2 Graduate school, Guangzhou University of Chinese Medicine, Guangdong Guangzhou, 510006 China; 3 Department of Pediatric, The Second Affiliated Hosptial of Zhejiang Chinese Medical University, Zhejiang Hangzhou, 310005 China; 4 Department of Orthopaedics & Traumatology, The First Affiliated Hospital of Zhejiang Chinese Medical University, Zhejiang Hangzhou, 310006 China

**Keywords:** Pelvic fracture, Iliosacral screw, Internal fixator (INFIX), Internal fixation, Minimally invasive

## Abstract

**Background:**

The commonly used technique for treating unstable pelvic fractures with sacroiliac screws and anterior internal fixator (INFIX) is prone to complications, such as injury to the pelvic vasculature and nerves, life-threatening bleeding, lateral femoral cutaneous neuritis, and wound infection. This study investigated the clinical effects of using a modified percutaneous iliosacral screw and INFIX technique for treating unstable pelvic fractures.

**Methods:**

A retrospective analysis of minimally invasive internal fixation using modified incision of an anterior-ring INFIX application combined with modified percutaneous iliosacral screw placement was performed for 22 cases of unstable pelvic fractures from January 2017 to December 2018. Based on the Tile classification, there were 4 type B1, 7 type B2, 5 type B3 and 6 type C1 injuries. Preoperatively, the length and orientation of the internal fixation were computer-simulated and measured. On postoperative day 3, pelvic radiographs and three-dimensional computed tomograms were used to assess fracture reduction and fixation. All patients were regularly followed up at 4 weeks, 12 weeks, 6 months, 12 months, 24 months and annually thereafter. Fracture healing, complications, visual analogue scale (VAS) scores, the quality of fracture repositioning and Majeed score were assessed during follow-up.

**Results:**

All patients were followed up for a mean of 25.23 ± 1.48 months. All fractures healed without loss of reduction and no patient showed evidence of delayed union or nonunion. Two years postoperatively, the mean VAS score was 0.32 ± 0.09 and the mean Majeed score was 94.32 ± 1.86.

**Conclusion:**

The modified percutaneous iliosacral screw technique increases the anterior tilt of the sacroiliac screw by shifting the entry point posteriorly to increase the safety of the screw placement. Downward modification of the INFIX incision reduces the risk of lateral femoral cutaneous nerve injury. This technique is safe, effective and well tolerated by patients.

## Introduction

The goal of treatment of pelvic fractures is to restore the anatomical integrity and stability of the anterior and posterior circumferential skeletal-ligamentous structures. Types B and C pelvic fractures according to the Tile classification [1] are unstable and require surgical treatment. Traditional incisional internal fixation has the shortcomings of surgical trauma, a long operation time, intraoperative bleeding, damage to important blood vessels and nerves, and difficult postoperative rehabilitation.

Percutaneous iliosacral screws can be combined with anterior internal fixators (INFIXs) in a minimally invasive procedure for unstable pelvic fractures. Biomechanical [2] and clinical studies [3, 4] have demonstrated the effectiveness of this procedure. However, we encountered some problems in clinical practice. First, INFIX may irritate the lateral femoral cutaneous nerve [3–5]. The most commonly used position (supine or prone) for iliosacral screw fixation is also problematic. The Kirschner guidewire for the sacroiliac screw is often blocked by the bed surface, making it difficult to enter the needle in the supine position. In the prone position, unstable pelvic fractures may occur under abnormal stress because the anterior ring is not fixed. Avoiding these above problems is very important in clinical practice.

We retrospectively analysed the clinical outcomes of unstable pelvic fractures treated using a combination of modified percutaneous iliosacral screw placement and a modified incision in the anterior INFIX technique.

### Patients and methods

Following institutional review board approval, 22 patients with unstable pelvic fractures admitted between January 2017 and December 2018 were included, with the injuries stemming from 17 traffic accidents and 5 falls from heights. There were 15 males and 7 females aged 47.18 ± 11.46 years (range: 24–65 years). Preoperative orthogonal exit and entrance pelvic radiographs and three-dimensional (3D) computed tomograms (CTs) were obtained. Based on the Tile classification, there were 4 type B1, 7 type B2, 5 type B3, and 6 type C1 cases (Table 1). At the time of admission, all patients had undergone pelvic pocket or external fixation (EXFIX) brace fixation. In type C patients, bone traction of one-sixth of the body weight was applied on the affected femoral condyle. The time from injury to operation was 10.00 ± 2.9 days (range: 6–16 days).

**Table 1:** Patient characteristics, operative details, and outcomes

Case no.	Sex	Age (years)	Type of pelvic fracture (Tile classification)	Mode of injury	Associated injuries	Duration from injury to surgery (days)	Duration for percutaneous iliosacral screw fixation (min)	Duration for INFIX application (min)	Total procedure duration (min)	Blood loss (mL)	Time of INFIX removal (months)	Follow-up duration (months)	Complications	Majeed score at 2 years after surgery	VAS at 2 years after surgery
1	Female	37	B2	Fall	Tibial plateau fracture	10	33	23	56	120	6	24	Nil	96	1
2	Male	48	B2	Traffic accident	Ankle fracture	9	32	26	58	100	9	23	Nil	97	0
3	Female	62	C1	Traffic accident	Proximal humeral fracture	12	42	36	78	180	12	25	Nil	94	1
4	Male	65	B1	Traffic accident	Bladder rupture	14	45	31	76	160	6	27	Lateral femoral cutaneous nerve injury	92	1
5	Male	54	B2	Traffic accident	Kidney contusion	7	38	34	72	140	4	26	Nil	91	0
6	Male	47	C1	Traffic accident	Brain injury	12	32	35	67	140	6	25	Nil	93	0
7	Female	60	B1	Fall	Lumbar transverse process fracture	6	35	36	71	150	5	26	Nil	95	1
8	Male	58	B3	Traffic accident	Brain injury	6	46	32	78	160	10	25	Nil	94	0
9	Male	49	C1	Traffic accident	Rib fractures, pleural effusion	10	49	33	82	180	9	24	Superficial incision-site infection	92	1
10	Male	24	B2	Traffic accident	Brain injury	7	32	25	57	100	7	26	Nil	93	0
11	Male	32	B2	Fall	Brain injury, cervical fracture	9	31	28	59	100	8	25	Nil	94	0
12	Female	46	B1	Traffic accident	Femoral fracture	10	35	24	59	80	6	24	Nil	92	1
13	Male	34	B2	Traffic accident	Lumbar fracture	8	28	23	51	90	7	28	Nil	93	0
14	Male	45	B3	Traffic accident	Rib fracture, thoracic vertebra fracture	9	30	25	55	90	7	25	Nil	95	0
15	Male	47	C1	Fall	Intertrochanteric fracture	12	34	32	66	120	12	22	Nil	92	1
16	Female	36	B3	Traffic accident	Proximal humeral fracture	10	35	26	61	100	8	26	Nil	96	0
17	Male	42	C1	Traffic accident	Rib fractures, hemopneumothorax	16	42	35	77	100	12	25	Nil	97	0
18	Female	34	B2	Traffic accident	Humeral fracture	11	31	24	55	90	9	24	Nil	96	0
19	Male	53	B3	Traffic accident	Urethral rupture	15	29	25	54	110	6	25	Nil	95	0
20	Male	62	B1	Traffic accident	Rib fractures	6	32	26	58	110	7	27	Nil	96	0
21	Female	41	B2	Fall	Splenic rupture	7	37	24	61	100	8	28	Nil	97	0
22	Male	62	C1	Traffic accident	Tibial fracture	14	38	27	65	100	8	25	Nil	95	0

The inclusion criteria were ① 18 < age < 70 years and ② Tile type B or C1 fractures.

The exclusion criteria were ① open pelvic fractures requiring emergency management, ② severe osteoporosis, ③ soft tissue infection at the expected nail placement site, ④ internal fixation precluded by concomitant thoracic and abdominal injuries, and ⑤ hemodynamic instability.

### Surgical technique

#### Analog measurement

**Fig. 1:**
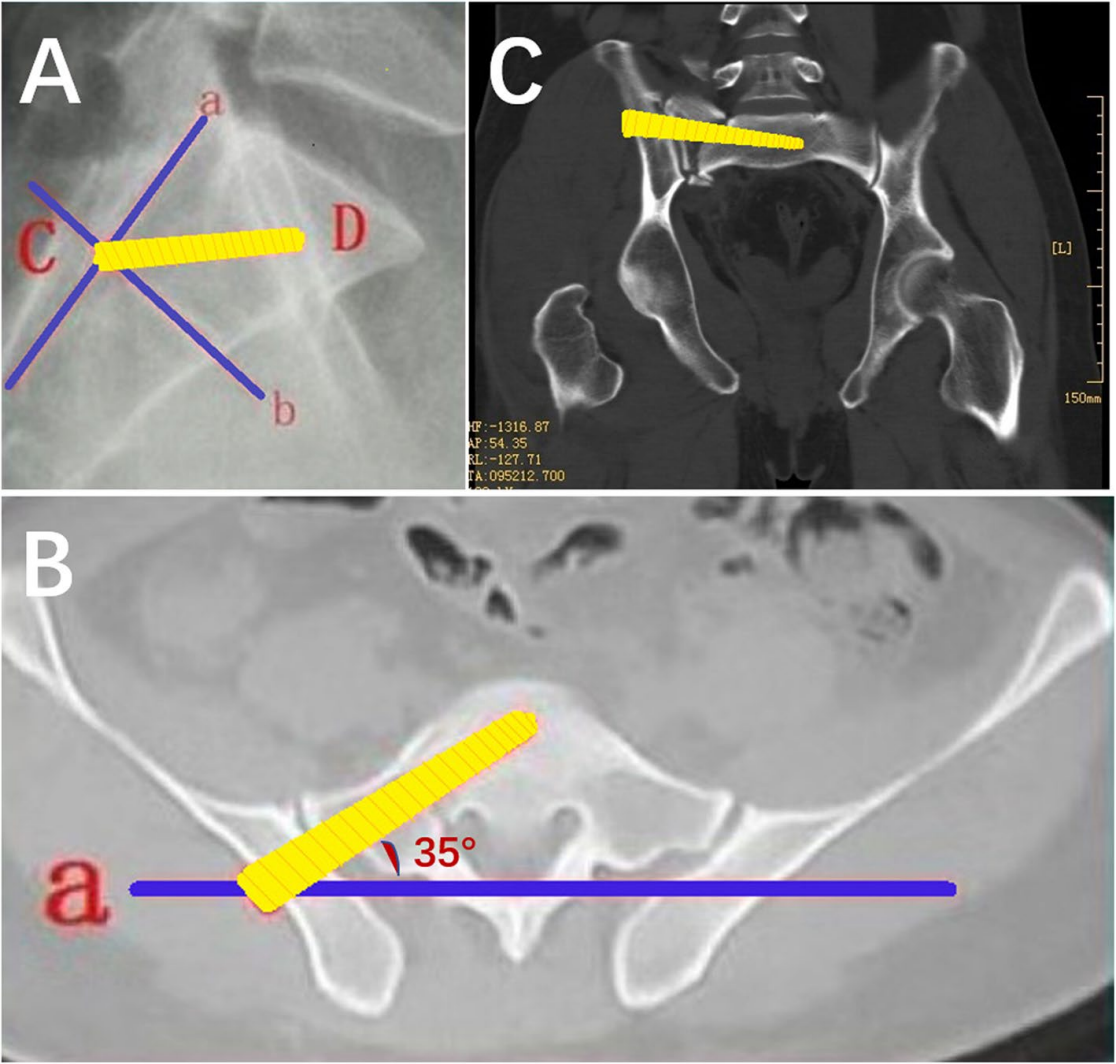
51-year-old male with Tile type B2 pelvic fracture. **A**: Lateral radiograph shows the nail placement point at the posterior edge of the spinal canal; **B**: transverse CT shows that the nail placement point was displaced from the anterior to the posterior edge of the spinal canal and was tilted forward by about 35°. **C**: Coronal CT shows that the screw was oriented perpendicular to the sacral fracture line and located anteriorly about 0.5 cm from the anterior edge of the S1 vertebral body

Iliosacral screw placement direction was predetermined based on radiographs and 3D CTs (Fig. 1).

#### Surgical positioning and fracture reduction

All surgeries were performed under general anaesthesia in a supine position on the uninjured side. The operator applied traction on the affected limb, while an assistant stabilised the axilla, to reduce vertical displacement of the pelvis. Pelvic inlet and outlet fluoroscopy were then performed to confirm the reduction.

### Surgical procedure

#### Iliosacral screw fixation

**Fig. 2:**
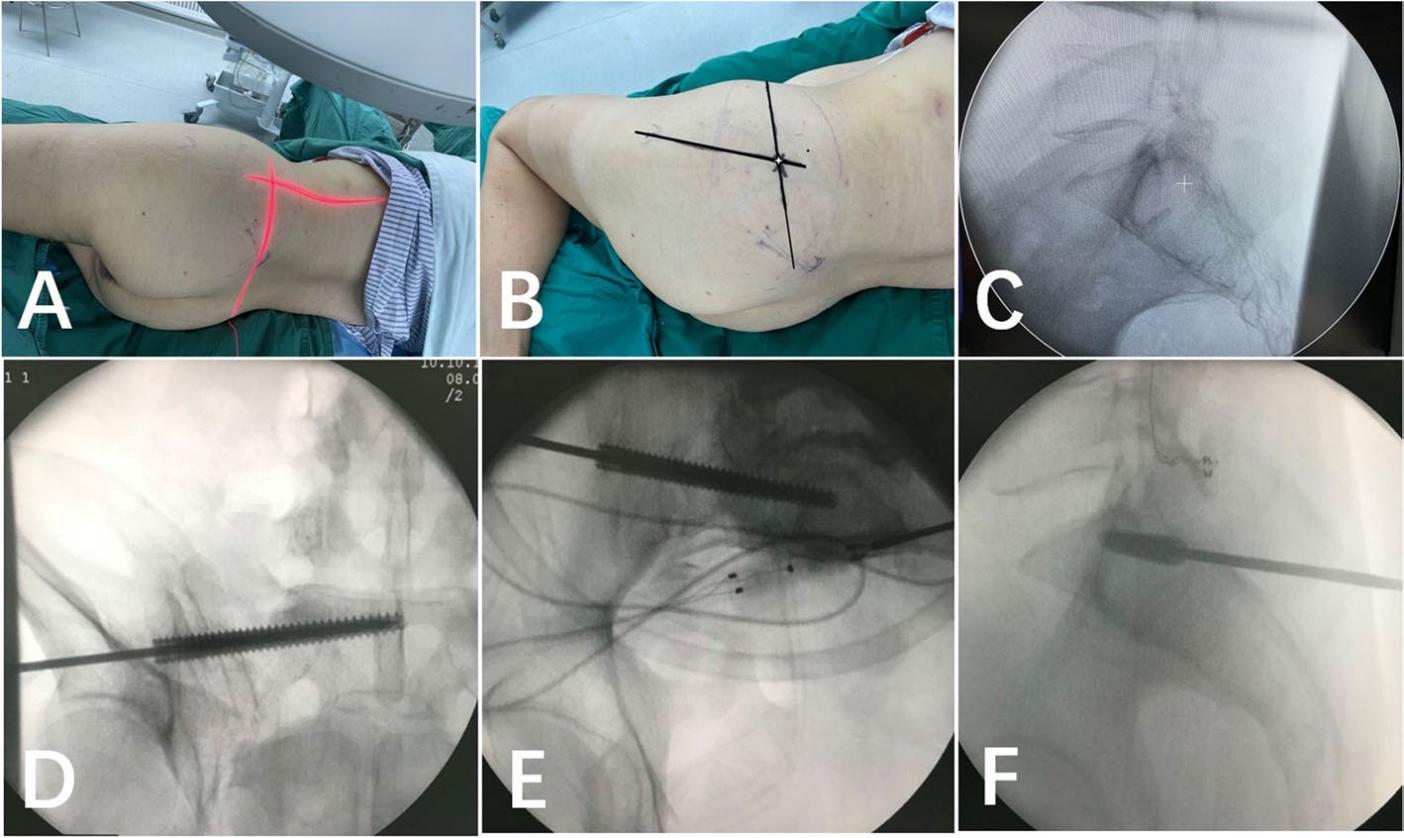
**A**: C-arm fluoroscope unit laser localisation identified the body entry point of the iliosacral screw guide needle at the intersection of the line connecting the anterior superior iliac spine with the posterior inferior iliac spine and the upward extension of the femoral stem; **B**: marking of the body entry point; **C**: the entry point of the guide needle was located at the intersection of the S1/2 endplate extension with the posterior wall of the sacral canal (+); **D**: pelvic outlet position indicated by the guide pin between the S1 superior endplate and the S1 sacral foramen; **E**: pelvic entry position indicated by the guide pin within 0.5 cm of the anterior edge of S1 before the midpoint of the section; **F**: lateral view of the sacrum showing the iliosacral screw entry point at the posterior edge of the spinal canal

The patients were placed in the lateral decubitus position; the entry point of the iliosacral screw guide was determined and marked using C-arm fluoroscope laser positioning. The patients were then placed in a supine position with 3–5 cm of buttock padding on the affected side, disinfected and draped. Pelvic entry- and exit-point fluoroscopy was performed to determine the correct orientation of the guide pin, and a 7-mm variable pitch cannulated screw was screwed in (Fig. 2). The positions of patients with bilateral sacroiliac joint dislocation, such as Tile type B2 and B3 fractures, were changed after unilateral screw fixation to enable fixation of the other side.

#### INFIX fixation

**Fig. 3:**
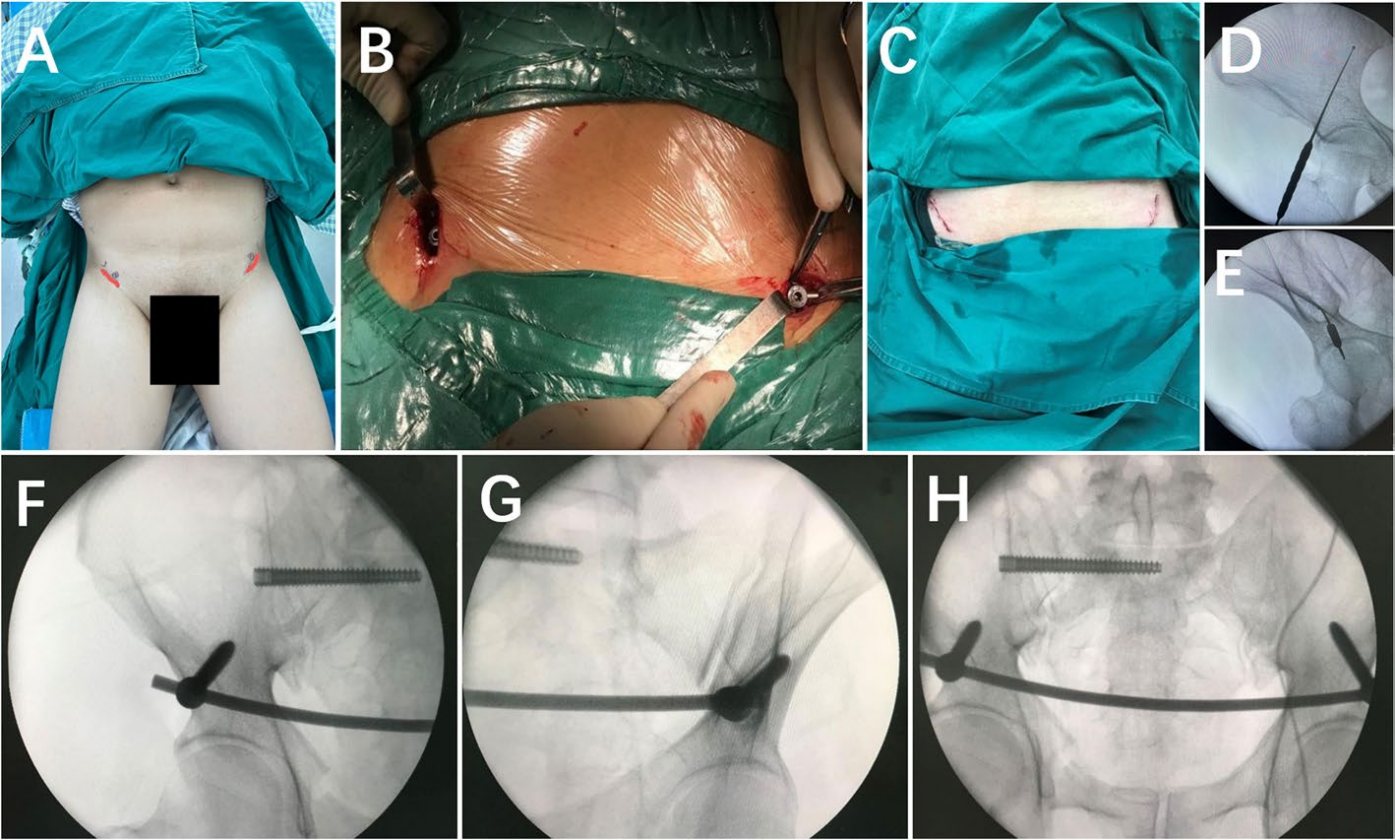
**A**: Schematic diagram of the INFIX incision; **B**: intraoperative screw placement; **C**: postoperative sutured incision; **D**, **E**: intraoperative fluoroscopy identifies the position of the guide pin in the ‘teardrop’ of the internal and external iliac plates; **F–H**: fluoroscopy shows good position of the INFIX.

An approximately 3 cm incision was made at 1 cm below the anterior inferior iliac spine (AIIS) bilaterally. A guide needle was inserted along the AIIS towards the posterior inferior iliac spine into the ‘teardrop,’ and its position was confirmed using fluoroscopy. A subcutaneous tunnel was created at the level of the pubic symphysis above the fascia layer under the skin, and the anterior pelvic ring fracture was reduced and fixed after installing a spinal rod (Fig. 3).

#### Postoperative treatment

**Fig. 4:**
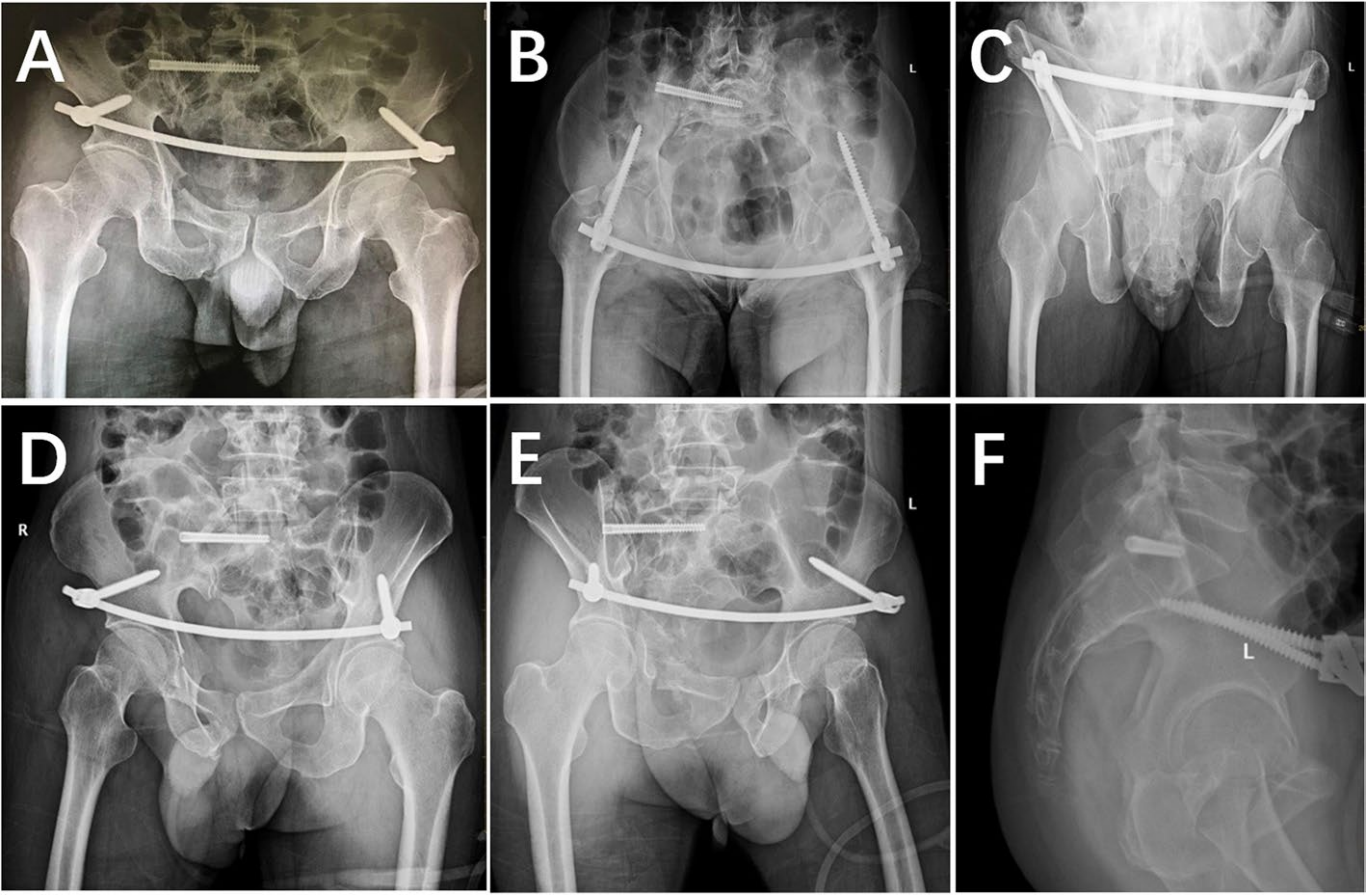
**A**–**F**: Postoperative day 3 radiograph showing well-reduced anterior and posterior rings with firm and well-positioned internal fixation

**Fig. 5:**
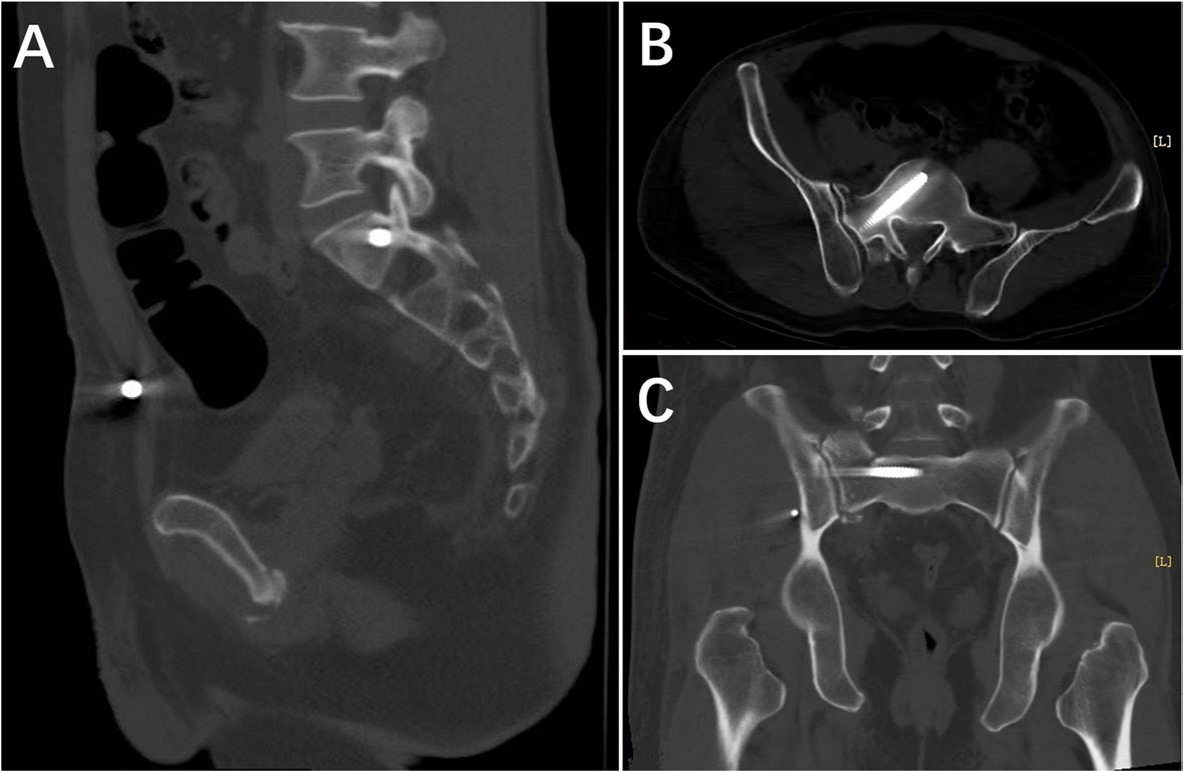
**A**: Postoperative day 3 sagittal CT showing the iliosacral screw position; **B**: transverse section showing sacral-fracture reduction; **C**: coronal section showing fracture reduction and the iliosacral screw position

Quadriceps exercises were started on postoperative day 2. Hip and knee flexion exercises were completed within 1 week. Patients were instructed to walk with toe contact using a walker, starting at 4 weeks postoperatively, and to start partial weight bearing at 8 weeks and full weight bearing at 3 months postoperatively. Radiographs (Fig. 4) and CTs (Fig. 5) were reviewed on postoperative day 3. All patients were regularly followed up at 4 weeks, 12 weeks, 6 months, 12 months, 24 months and annually thereafter. Fracture healing, complications, visual analogue scale (VAS) scores, Matta criteria [6] (reductions graded as excellent: ≤ 4 mm; good: 5–10 mm; fair: 10–20 mm; and poor: ˃ 20 mm, based on the maximal displacement measured on three standard pelvic views) and the Majeed score [7] for pelvic function were assessed during follow-up. The INFIX was removed at 6–12 months postoperatively with a mean time of 7.82 ± 2.22 months, but the iliosacral screw was not removed unless there were neurological symptoms.

### Statistical analysis

Statistical analysis was performed using SPSS software (ver. 26.0; IBM Corp., Armonk, NY, USA). The data for each group are expressed as the mean ± SD. Mortality, complications, missing data, follow-up time, and functional assessment results were analysed.

## Results

The patients were followed for a mean of 25.23 ± 1.48 months. There were no deaths during follow-up. All fractures healed without loss of reduction and no patient showed evidence of delayed union or nonunion. The mean intraoperative blood loss was 119.09 ± 30.38 mL; the mean operative time was 64.36 ± 9.35 min, including 35.73 ± 5.81 min and 28.64 ± 4.67 min for iliosacral screw and INFIX placement, respectively. One patient presented with lateral femoral cutaneous nerve injury, which gradually improved with mecobalamin therapy. Another patient developed a superficial infection at the anterior ring nail opening, which was treated using wound dressings. The VAS score recovered to 3.12 ± 0.83 at 6 months and 0.32 ± 0.09 at 2 years postoperatively. The mean Majeed score was 94.32 ± 1.86 at 2 years postoperatively (Table 1).

## Discussion

Surgical techniques, such as those using closed-replacement pubic branch screws, iliosacral screws, spinal arch nail rod systems, and anterior ring INFIX systems, have been used in recent years to treat pelvic ring injuries [8–10]. However, the optimal fixation method is still controversial [9, 10]. INFIX and iliosacral screw approaches are currently the methods of choice for minimally invasive fixation of anterior and posterior rings [11].

### Indications of INFIX use and iliosacral screw fixation

Modified percutaneous iliosacral screw and anterior INFIX techniques are primarily indicated for unstable pelvic fractures without sacral plexus injuries [3, 4]. Vaidya et al. [11] proposed anterior pelvic ring injuries combined with obesity as the best indication for INFIX use. The surgical indications were later expanded to include type B injuries [12] according to the Tile classification, type 61-B and C injuries [3] according to the AO/OTA classification and injuries coded as LC, APC, VS and CMI according to the Young-Burgess classification [5]. Only Tile B and C1 fractures were included in this study due to the limited number of cases and the fact that open pelvic fractures were not included. A systematic review reported an infection rate of 5.4% (24/445 patients) when using INFIX for closed injuries [13]. The risk of INFIX infections is higher for open injuries than closed injuries. Open injuries are often accompanied by hemodynamic instability and require EXFIX as soon as possible, although Vaidya et al. [14] reported four cases in which Gustilo III open pelvic fractures were treated successfully with EXFIX/INFIX. Primary debridement and EXFIX fixation and secondary INFIX were performed in two cases. The other two patients underwent primary debridement and INFIX fixation. However, the exposed INFIX implants had a higher infection rate compared with EXFIX. The INFIX technique may be the choice of fixation in hemodynamically stable and uninfected patients.

### Surgical techniques

Complications of INFIX use include anterolateral femoral cutaneous nerve injuries and femoral nerve compression [15–17]. The reported incidence of anterolateral femoral cutaneous nerve injury is 29.7% (27/91 patients) [18]. This may be transient [18] or persistent [19], and it may resolve following INFIX removal [20]. The traditional INFIX involves a 2–3 cm longitudinal incision at the groin crease, centred on the AIIS [11]. We moved the AIIS incision 1 cm inferiorly to avoid anterolateral femoral cutaneous nerve injury [18–20]. The lateral femoral cutaneous nerve remains above the incision and requires gentle manipulation and traction intraoperatively. Retaining the threads of the 1–1.5 cm INFIX screw outside the bone reduces the risk of femoral nerve compression by the spinal rod. In this study, 1/22 patients (4.5%) experienced postoperative unilateral lateral femoral cutaneous nerve injury, presumably the result of intraoperative traction to reveal the anterior inferior iliac spine, which resolved in 4 weeks.

**Fig. 6:**
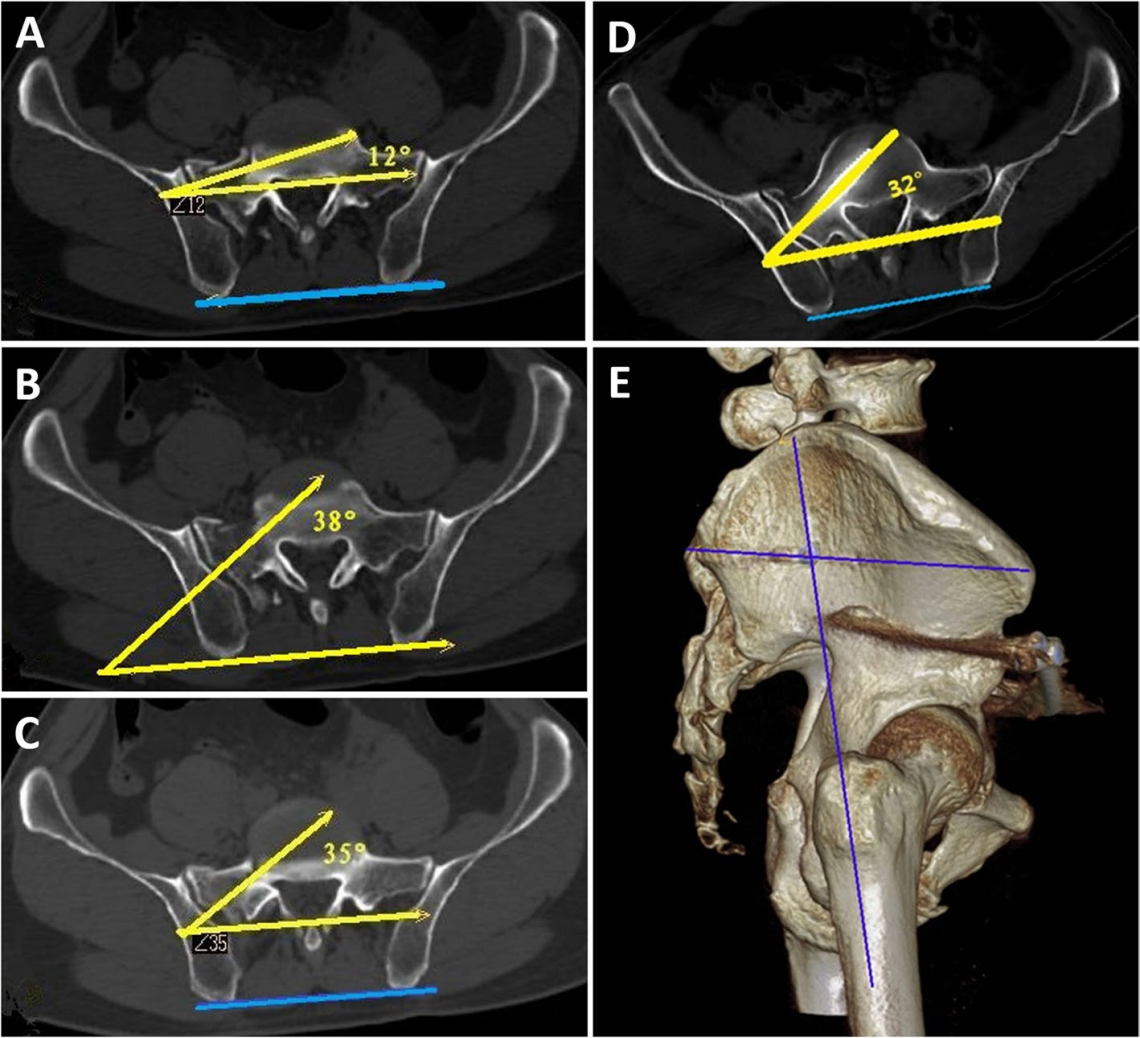
**A**: Mendel et al. [27] used the anterior edge of the spinal canal as the nail entry point with an angle of 12°; **B**: Hou et al. [28] used an anterior tilt angle of 38°; **C**: preoperative nail entry point angle of 35° with the posterior edge of the spinal canal; **D**: postoperative nail entry point angle of 32° with the posterior edge of the spinal canal; **E**: 3D CT reconstruction confirmed the location of the nail entry point at the intersection of the line connecting the anterior superior iliac spine and posterior inferior iliac spine with the upward extension of the femoral stem

Complications of iliosacral screw placement include implant failure, infection, and injury to the superior gluteal artery, iliac vessels, and lumbosacral nerves [21–25]. Deviations in the iliosacral screw placement direction increase the risk of neurovascular injuries. The incidence of screw malposition may be as high as 24% [26]. Mendel et al. [27] tilted the surgical bed at 12° to the healthy side and effectively avoided these injuries by horizontal placement of the guide needles. Hou et al. [28] determined an optimal anteversion angle of 38.3 ± 1.9° in cadaveric experiments. We measured an anterior screw angle of 34.7 ± 2.3° (range: 32–37°) in the sacrum (Fig. 6). The angle change was due to a posterior shift in the entry point of the guide needle from the anterior to the posterior edge of the sacral canal, which is positioned on the body surface at the intersection of the line joining the anterior superior iliac spine and the posterior inferior iliac spine with the upward extension of the femoral stem. This approach increased the range of adjustable angles for the screws in the bone and the range of safe access. This is the main reason for the lack of complications, as well as the small number of included cases. At the same time, the sacroiliac screws were placed in the lateral decubitus position. This modified position effectively avoids the obstruction of the bed surface that occurs in the supine position, and the improper stress of the anterior pelvic ring seen in the prone position.

### Advantages and disadvantages of using INFIXs and iliosacral screws

The INFIX exhibits good resistance strength against axial shifting and separation [12], and its strength is 23% greater than with EXFIX [5, 11]. Partial weight-bearing exercises can be performed early in the postoperative period by patients treated with INFIXs. Iliosacral screws are effective for fixing posterior pelvic ring fractures and dislocations. Most surgeons now use one or more screws, especially for sacral fractures or trans-sacral screws, which are stronger. Our study included 22 cases, including 4, 7, 5, and 8 type B1, B2, B3, and C1 cases, respectively. We used only one 7-mm variable pitch cannulated screw to fix the sacroiliac joint, and achieved satisfactory clinical results and fracture healing. Honey et al. confirmed that posterior arch fixation of the pelvic ring with one sacroiliac screw, along with beside anterior arch fixation in an unstable pelvis fracture, is sufficient fixation to maintain the stability required for complete fracture union [29]. It effectively resists shear and torsional forces following pelvic ring injuries with combined anterior- and posterior-ring minimally invasive fixation. In addition, early functional exercises stimulate growth and ultimately lead to optimal fracture healing and functional recovery.

In this study, the mean intraoperative blood loss and mean operative time were similar to the findings by Shetty et al. and Liu et al. [3, 4]. Patients recovered rapidly without complications, such as wound necrosis or decubitus ulcers. The quality of Matta repositioning at the end of follow-up in this study was rated as excellent. All patients exhibited evidence of fracture healing on imaging. The mean Majeed score of pelvic function was 94.32 ± 1.86 at 2 years postoperatively. Compared to conventional open reduction and internal fixation, this procedure has the advantages of less surgical trauma, reduced intraoperative bleeding, a shorter operative time, fewer postoperative complications, and faster recovery.

## Limitations

The limitations of our study were the retrospective design, small sample size, lack of Tile type C2 and C3 cases, absence of a control group and relatively short postoperative follow-up period. Large, multicentre, prospective, randomised controlled studies will be required in the future.

## Conclusion

The modified percutaneous iliosacral screw and anterior INFIX technique can achieve effective fixation and excellent clinical outcomes in unstable pelvic ring injuries. It is a safe and effective treatment with the advantage of being well tolerated by patients.

## Data Availability

The datasets analysed in this study are available from the corresponding author on reasonable request.

## Abbreviations

INFIX: Internal fixator
VAS: Visual analogue scale
EXFIX: External fixation
AIIS: Anterior inferior iliac spine
